# Isolated Pulmonary Arteriovenous Fistula-Related Embolic Stroke With Large Vessel Occlusions Mimicking Artery-to-Artery Embolism: A Case Report

**DOI:** 10.7759/cureus.88555

**Published:** 2025-07-22

**Authors:** Zhang Meixia, Pan Xiaoling, Chen Hongfang

**Affiliations:** 1 Neurology, Affiliated Jinhua Hospital, Zhejiang University School of Medicine, Jinhua, CHN

**Keywords:** artery-to-artery embolism, embolic stroke, pulmonary arteriovenous fistula, recombinant tissue plasminogen activator, stroke

## Abstract

Isolated pulmonary arteriovenous fistula (PAVF) leading to paradoxical embolism and stroke is rare, particularly in cases involving large vessel occlusions. Here, we present the case of a 69-year-old female with occlusion of the M2 segment of the middle cerebral artery (MCA) and stenosis of the common carotid artery (CCA) caused by PAVF, which mimicked artery-to-artery embolism. CT angiography revealed occlusion of the left M2 segment of the MCA and stenosis of the CCA. After administration of recombinant tissue plasminogen activator, both the left MCA occlusion and CCA stenosis were completely recanalized. Transthoracic contrast echocardiography revealed a significant right-to-left shunt both at rest and during the Valsalva maneuver, while chest CT angiography indicated the presence of PAVF in the lower lobe of the right lung. The anticoagulant medication rivaroxaban (15 mg) was administered to prevent the recurrence of ischemic stroke. Pulmonary arterial angiography confirmed the diagnosis of PAVF, and PAVF embolization using coils was successfully performed. At the one-year follow-up, the patient had no stroke recurrence. PAVF is a potentially fatal but treatable disease. Even in patients with large vessel occlusions, it is essential to consider PAVF as a rare underlying cause. The mechanism of PAVF-related stroke might be mistaken for artery-to-artery embolism.

## Introduction

Pulmonary arteriovenous fistulae (PAVFs) are pathologic low-resistance, high-flow conduits between a pulmonary artery and vein without intervening capillaries. These fistulae can cause paradoxical embolism and lead to acute ischemic stroke (AIS). Approximately 70% of PAVFs are multiple, congenital, and recognized as significant complications of hereditary hemorrhagic telangiectasia (HHT) [1]. Isolated PAVF that induces paradoxical embolism leading to stroke is rare, particularly in cases involving large vessel occlusions. As PAVFs may also be fatal but can be treated through embolization, early diagnosis is critical. In artery-to-artery embolism, imaging shows small cortical infarcts or a single territory infarct in the area supplied by the relevant intracranial or extracranial artery atherosclerosis [2]. Here, we present the case of a 69-year-old female patient who experienced M2 segment occlusion of the middle cerebral artery (MCA) along with stenosis of the common carotid artery (CCA), which was initially diagnosed as artery-to-artery embolism related to large arterial atherosclerosis. On examination, we found the embolism to be resulting from a paradoxical embolism associated with PAVF.

This article was previously posted to the Research Square preprint server on February 15, 2024.

## Case presentation

**Figure 1:**
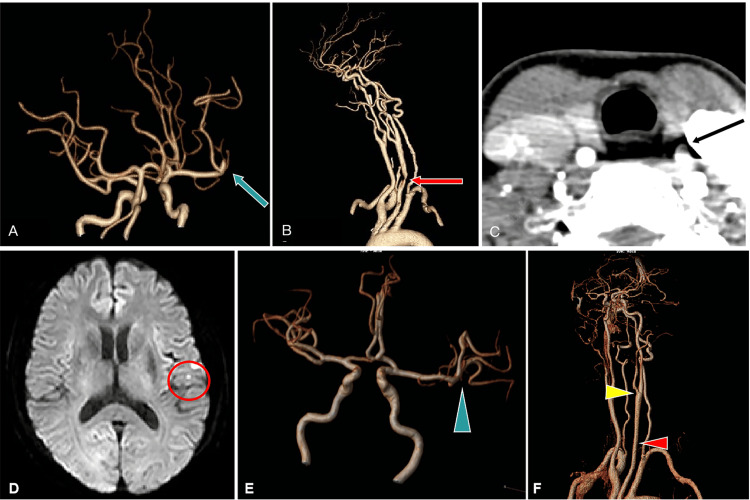
CT and MRI findings. Pre-thrombolysis reconstructed CT angiography (CTA) demonstrating occlusion of the left M2 segment of the middle cerebral artery (MCA) (A, blue arrow) and stenosis of the common carotid artery (CCA) (B, red arrow). Pre-thrombolysis CTA showing a thrombus-like shadow of the common carotid artery (CCA) (C, black arrow). Post-thrombolysis diffusion-weighted MRI revealing a small ischemic lesion in the front lobe (D, red circle). Post-thrombolysis magnetic resonance angiography (MRA) showing the occlusion of the left MCA (E, blue arrowhead). Post-thrombolysis MRA showing the stenosis of left CCA (red arrowhead) was successfully recanalized, and a filling defect can be observed in the left external carotid artery (F, yellow arrowhead).

A 69-year-old woman arrived at the neurology department of our hospital with sudden-onset aphasia. The symptoms had begun one hour earlier and resolved within 30 minutes. However, 10 minutes later, the aphasia recurred, with a National Institutes of Health Stroke Scale (NIHSS) score of 5. Non-contrast CT of the head revealed no bleeding, while CT angiography (CTA) indicated left M2 segment occlusion of the MCA (Figure 1A) and stenosis of the CCA (Figures 1B, 1C). The patient received intravenous thrombolysis therapy using recombinant tissue plasminogen activator 93 minutes after symptom onset, which resulted in complete symptom resolution with an NIHSS score of 0. No additional endovascular therapy was conducted. Upon admission, she was afebrile with normal vital signs, and a general examination was performed. She did not smoke, consume alcohol, or use illicit drugs. No family members had a history of epistaxis or gastrointestinal bleeding. There was no history of other vascular risk factors, including hypertension, diabetes mellitus, and hyperlipidemia. There were no symptoms of epistaxis or gastrointestinal bleeding before this admission. She had a history of breast fibroadenoma excision three years ago, invasive adenocarcinoma treated with lung resection two years ago, and lower limb aneurysm embolization. The following day, a detailed head MRI revealed a small infarct in the left frontal lobe (Figure 1D), while magnetic resonance angiography demonstrated complete recanalization of the M2 segment of the MCA (Figure 1E). One week later, there were no filling defects observed in the CCA, but a filling defect was noted in the left external carotid artery (Figure 1F).

Routine blood tests, along with liver and kidney function assessments, electrolytes, coagulation profile, glycosylated hemoglobin levels, antiphospholipid syndrome antibodies, autoimmune antibody spectrum analysis, tumor markers, and immunoglobulins revealed no abnormalities. Lipid levels and homocysteine were within the normal ranges. A 24-hour dynamic electrocardiogram showed no atrial flutter or fibrillation, and Doppler ultrasound demonstrated minimal regurgitation in the aortic and tricuspid valves. Abdominal ultrasound examination of the liver, gallbladder, pancreas, spleen, urinary system, and breast revealed no abnormalities. Thyroid ultrasound identified a nodule in the right lobe. The pelvic examination revealed a postmenopausal uterus containing intracavitary fluid along with a cystic lesion in the left adnexal region. No obvious mass was detected in the right adnexal region. Doppler ultrasound of the lower limbs performed on the third day of admission suggested a possible deep vein thrombosis in the left leg.

**Figure 2:**
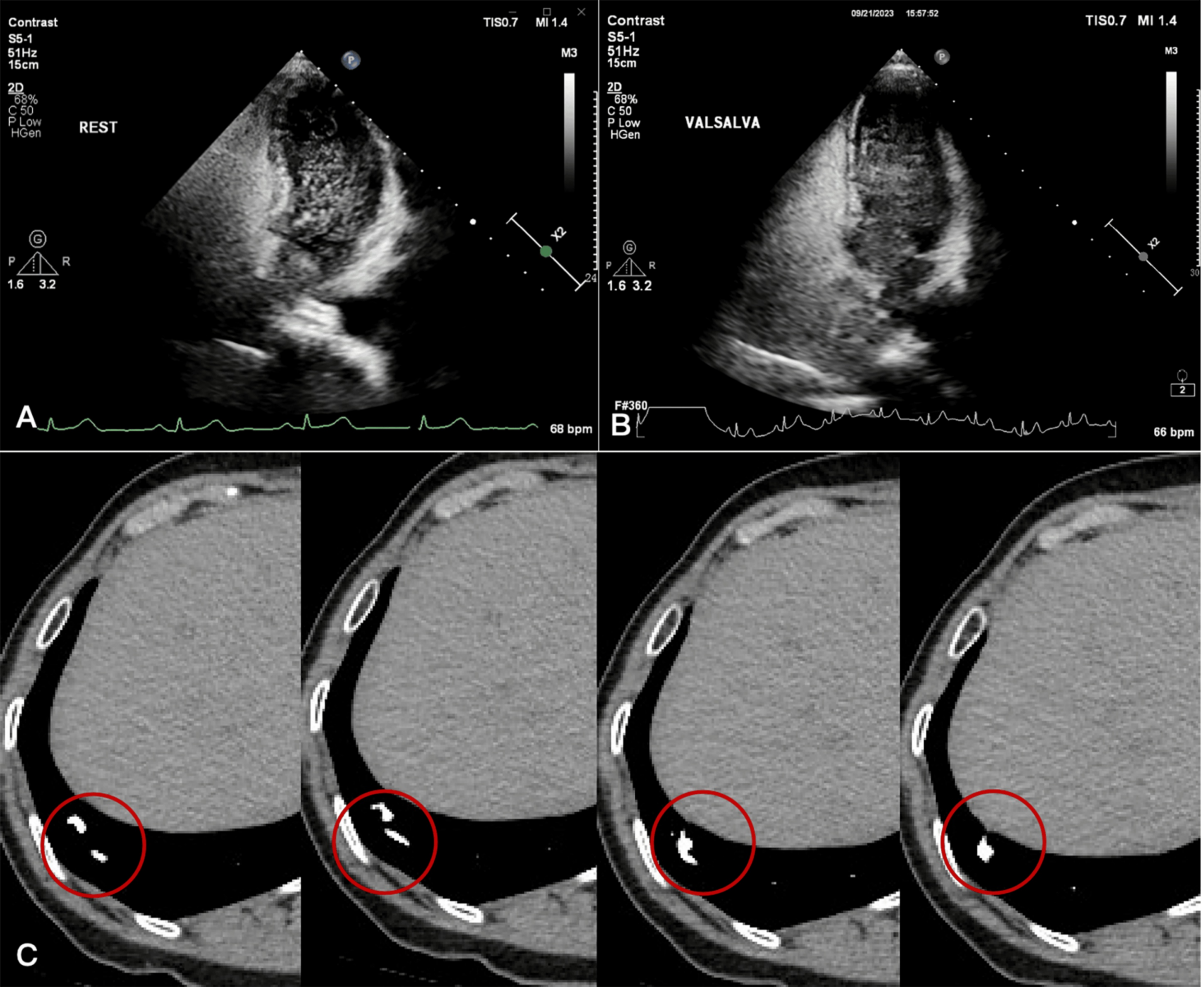
Transthoracic contrast echocardiography image and CT angiography of the chest. Transthoracic contrast echocardiography revealing a significant right-to-left shunt both at rest (A) and during the Valsalva maneuver (B). (C) CT angiography of the chest suggesting the formation of a pulmonary arteriovenous fistula in the lower lobe of the right lung (red circle).

**Figure 3:**
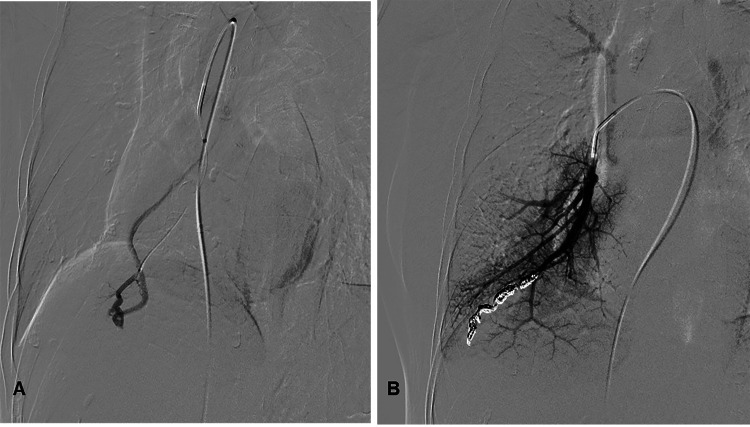
Pulmonary angiography. (A) Pretreatment angiography indicating the presence of a pulmonary arteriovenous fistula (PAVF) in the lower lobe of the right lung. (B) Post-treatment showing that the PAVF was embolized with coils.

Transthoracic contrast echocardiography revealed a significant right-to-left shunt both at rest (Figure 2A) and during the Valsalva maneuver (Figure 2B), raising the suspicion of PAVF. Subsequently, chest CTA indicated the presence of PAVF in the lower right lung (Figure 2C). The anticoagulant medication rivaroxaban (15 mg) was administered to prevent a recurrence of ischemic stroke. Three months later, the patient underwent selective right pulmonary arterial angiography, which confirmed the diagnosis of PAVF (Figure 3A). PAVF embolization using coils was successfully performed, resulting in the complete disappearance of shunt blood flow (Figure 3B) with no adverse and unanticipated events. After embolization therapy, aspirin was used to prevent the recurrence of ischemic stroke. No speech disorders were observed, and no strokes recurred during follow-up one year later through outpatient service.

## Discussion

PAVF is a rare vascular anomaly characterized by an abnormal connection between the pulmonary artery and vein without capillaries in the lungs [3], leading to various degrees of right-to-left shunt. A 2012 analysis using thoracic CT scanning showed a prevalence rate of 38/100,000, with the incidence being twice as common in women [4]. The incidence of stroke among PAVF patients with HTT has been reported to range from 9.3% to 70%, while data on isolated PAVF patients are limited [5,6]. Notably, PAVF is recognized as an important cause of stroke in young adults [7]. Another retrospective study reported that the ratio of PAVF in AIS was 0.02%, with the mean age of affected individuals being 57.5 years [8]. In other words, the initial diagnosis of PAVF is even rarer in elderly stroke patients.

PAVF-related ischemic strokes can be either cortical or subcortical, but PAVF very rarely leads to proximal large vessel occlusions. Only one case report described a proximal (M1-MCA) large vessel occlusion [9], while another reported an occlusion in the right M2 segment of the MCA [10]. In PAVF, recurrent endothelial injury (e.g., from dysplastic vasculature) or other molecular mechanisms of hypercoagulability implicated in different right-to-left shunts can promote local thrombus formation and subsequent embolization [7]. Once formed, a thrombus within or proximal to the PAVF is exposed to systemic pressures and flow dynamics that can facilitate its dislodgement and passage into the systemic arterial circulation, leading to paradoxical embolic events such as stroke. Recently, a case report detailed the utilization of optical coherence tomography to examine the internal structures of PAVF and identified the potential source of thrombus [11]. Further, one case report described a PAVF that mimicked vertebral artery dissection [12]. In this case, the thrombi in the M2 segment of the MCA were initially thought to be from the CCA.

At present, authoritative clinical practice guidelines for intravenous thrombolytic therapy in PAVF-related paradoxical cerebral embolism are lacking. The safety and clinical benefits of thrombolysis have not been established [3]. Moreover, there are no cohort studies or case series that describe the use of intravenous thrombolytics in AIS patients with PAVF, with only a few case reports documenting their use [7]. In a study analyzing data from 2005 to 2014 [8], patients with and without PAVF received intravenous thrombolytics at similar rates (5.9% versus 5.8%), but bleeding complications were not compared. In general, the potential for bleeding complications is a significant concern with thrombolytic therapy in PAVF patients due to the high risk of fatal pulmonary hemorrhage, epistaxis, and gastrointestinal bleeding associated with comorbid telangiectasia, especially in cases of HHT [13]. However, PAVF is often undiagnosed until patients present with an ischemic stroke [7], making it challenging to diagnose at the time of stroke onset in real-world scenarios. In our case, the patient received intravenous thrombolysis and achieved an excellent outcome without any bleeding complications. Therefore, thrombolytic therapy may be a viable option for patients with isolated PAVF within an appropriate time window.

For secondary prevention of stroke, no clear guidelines exist for optimal antithrombotic management for cerebral embolism due to PAVF. A recent study [8] found that patients with PAVF were approximately twice as likely to be on long-term anticoagulation after adjusting for multiple confounders. The most recent international guidelines for HHT management recommend that, when indicated, preventive antithrombotic regimens consist of either single antiplatelet or anticoagulant therapy, avoiding double antiplatelet therapy or combined antiplatelet and anticoagulant therapy[14]. The second stroke prophylactic measure for ischemic strokes caused by large artery atherosclerosis is dual antiplatelet therapy. In our case, as the patient also had deep vein thrombosis, we selected rivaroxaban as a secondary prevention strategy before embolization therapy. Following the successful embolization of the PAVF and in the absence of deep vein thrombosis, we transitioned to aspirin for secondary prevention.

Endovascular embolization therapy is the preferred treatment for PAVF [1,13]. This intervention effectively reduces risks associated with paradoxical emboli and symptoms exacerbated by right-to-left shunting and haemorrhage[3]. Embolization is considered a safe procedure when performed by experienced practitioners. Currently, it is recommended for all treatable PAVFs, regardless of feeding artery size, including in asymptomatic patients [1].

## Conclusions

We presented a rare case of new-onset embolic stroke in an elderly patient who was finally diagnosed with PAVF. Initially, the thrombus in the M2 segment of the MCA in this patient was easily thought to originate from stenosis of the ipsilateral CCA, making artery-to-artery embolism the primary consideration for etiology. After intravenous thrombolysis treatment, both the MCA-M2 occlusion and the CCA stenosis completely disappeared, suggesting that the initially considered CCA stenosis was likely to be a thrombus. In clinical practice, more attention should be paid when imaging shows small cortical infarcts or a single territory infarct in the area supplied by the relevant intracranial or extracranial artery stenosis, including re-examining the target artery. PAVF is a potentially fatal but treatable disease, and AIS may be the first presenting symptom. Even in patients with large vessel occlusions, it is essential to consider PAVF as a rare underlying cause. Finally, the mechanism of PAVF-related stroke might be mistaken for artery-to-artery embolism.

## References

[1] Müller-Hülsbeck S, Marques L, Maleux G, Osuga K, Pelage JP, Wohlgemuth WA, Andersen PE (2020). CIRSE standards of practice on diagnosis and treatment of pulmonary arteriovenous malformations. Cardiovasc Intervent Radiol.

[2] Gao S, Wang YJ, Xu AD, Li YS, Wang DZ (2011). Chinese ischemic stroke subclassification. Front Neurol.

[3] Shovlin CL, Condliffe R, Donaldson JW, Kiely DG, Wort SJ (2017). British Thoracic Society Clinical Statement on Pulmonary Arteriovenous Malformations. Thorax.

[4] Nakayama M, Nawa T, Chonan T (2012). Prevalence of pulmonary arteriovenous malformations as estimated by low-dose thoracic CT screening. Intern Med.

[5] Kjeldsen AD, Oxhøj H, Andersen PE, Green A, Vase P (2000). Prevalence of pulmonary arteriovenous malformations (PAVMs) and occurrence of neurological symptoms in patients with hereditary haemorrhagic telangiectasia (HHT). J Intern Med.

[6] Faughnan ME, Lui YW, Wirth JA, Pugash RA, Redelmeier DA, Hyland RH, White RI Jr (2000). Diffuse pulmonary arteriovenous malformations: characteristics and prognosis. Chest.

[7] Topiwala KK, Patel SD, Saver JL, Streib CD, Shovlin CL (2022). Ischemic stroke and pulmonary arteriovenous malformations: a review. Neurology.

[8] Topiwala KK, Patel SD, Pervez M, Shovlin CL, Alberts MJ (2021). Ischemic stroke in patients with pulmonary arteriovenous fistulas. Stroke.

[9] Inoue S, Fujita A, Kurihara E, Sasayama T (2023). Mechanical thrombectomy for acute paradoxical cerebral embolism due to pulmonary arteriovenous malformation: a case report and review of literature. Surg Neurol Int.

[10] Yassi N, Yan B, Dowling R, Mitchell PJ (2014). A rare cause of embolic stroke in hereditary hemorrhagic telangiectasia. J Stroke Cerebrovasc Dis.

[11] Song M, Dai H, Lu W, Meng X (2025). Thrombus rooting in the pulmonary arteriovenous fistula in a patient with cryptogenic stroke, a case report. BMC Neurol.

[12] Isogai M, Suzuki T, Kato S, Taniguchi Y, Hasegawa H, Oishi M, Fujii Y (2023). A case of paradoxical cerebral embolism due to pulmonary arteriovenous fistula mimicking vertebral artery dissection with Wallenberg syndrome. Cureus.

[13] Lin G, Jiang P, Lou M (2019). Thrombolysis in ischemic stroke patients with isolate pulmonary arteriovenous malformations. J Stroke Cerebrovasc Dis.

[14] Faughnan ME, Mager JJ, Hetts SW, Palda VA, Ratjen F (2021). Second international guidelines for the diagnosis and management of hereditary hemorrhagic telangiectasia. Ann Intern Med.

